# Involvement of CD4^+^ Foxp3^+^ Regulatory T Cells in Persistence of *Leishmania donovani* in the Liver of Alymphoplastic *aly/aly* Mice

**DOI:** 10.1371/journal.pntd.0001798

**Published:** 2012-08-21

**Authors:** Saruda Tiwananthagorn, Kazuya Iwabuchi, Manabu Ato, Tatsuya Sakurai, Hirotomo Kato, Ken Katakura

**Affiliations:** 1 Laboratory of Parasitology, Graduate School of Veterinary Medicine, Hokkaido University, Sapporo, Japan; 2 Department of Veterinary Biosciences and Veterinary Public Health, Faculty of Veterinary Medicine, Chiang Mai University, Chiang Mai, Thailand; 3 Department of Immunology, Kitasato University School of Medicine, Kanagawa, Japan; 4 Department of Immunology, National Institute of Infectious Diseases, Tokyo, Japan; Yale School of Public Health, United States of America

## Abstract

Visceral leishmaniasis (VL) is a chronic and fatal disease in humans and dogs caused by the intracellular protozoan parasites, *Leishmania donovani* and *L. infantum* (*L. chagasi*). Relapse of disease is frequent in immunocompromised patients, in which the number of VL cases has been increasing recently. The present study is aimed to improve the understanding of mechanisms of *L. donovani* persistence in immunocompromised conditions using alymphoplastic *aly/aly* mice. Hepatic parasite burden, granuloma formation and induction of regulatory T cells were determined for up to 7 months after the intravenous inoculation with *L. donovani* promastigotes. While control *aly/+* mice showed a peak of hepatic parasite growth at 4 weeks post infection (WPI) and resolved the infection by 8 WPI, *aly/aly* mice showed a similar peak in hepatic parasite burden but maintained persistent in the chronic phase of infection, which was associated with delayed and impaired granuloma maturation. Although hepatic CD4^+^Foxp3^+^ but not CD8^+^Foxp3^+^ T cells were first detected at 4 WPI in both strains of mice, the number of CD4^+^Foxp3^+^ T cells was significantly increased in *aly/aly* mice from 8 WPI. Immunohistochemical analysis demonstrated the presence of Foxp3^+^ T cells in *L. donovani*–induced hepatic granulomas and perivascular neo-lymphoid aggregates. Quantitative real-time PCR analysis of mature granulomas collected by laser microdissection revealed the correlation of *Foxp3* and *IL-10* mRNA level. Furthermore, treatment of infected *aly/aly* mice with anti-CD25 or anti-FR4 mAb resulted in significant reductions in both hepatic Foxp3^+^ cells and parasite burden. Thus, we provide the first evidence that CD4^+^Foxp3^+^ Tregs mediate *L. donovani* persistence in the liver during VL in immunodeficient murine model, a result that will help to establish new strategies of immunotherapy against this intracellular protozoan pathogen.

## Author Summary

The protozoan parasite *Leishmania donovani* is the causative agent of visceral leishmaniasis (VL) with a variety of outcomes ranging from asymptomatic to fatal infection. In the last decade, an increasing number of VL cases in immunocompromised conditions have been reported. Loss of the control of parasite persistence causes relapse of the disease in these patients. To clarify why parasite persistence and disease are caused in an immunocompromised condition, we examined *L. donovani* infection in alymphoplastic *aly/aly* mice that completely lack lymph nodes and have disturbed spleen architecture. Although parasites grew in the liver of *aly/+* mice for the first 4 weeks post infection (WPI) and parasites were eliminated by 8 WPI, we found that parasites persisted in the liver of *aly/aly* mice with the ineffective of granuloma formation to kill the parasites. These *aly/aly* mice showed significant increases in CD4^+^Foxp3^+^ regulatory T cells in the liver. Consequently, we treated infected mice with anti-CD25 or anti-FR4 mAb to inhibit the function of Tregs, and found significant reductions in both hepatic Foxp3^+^ cells and parasite burden. These results clearly demonstrated for the first time that the expansion of CD4^+^Foxp3^+^ Tregs is involved in hepatic *L. donovani* persistence in immunodeficient murine model.

## Introduction

Visceral leishmaniasis (VL) is a chronic and fatal disease caused by the intracellular protozoan parasites *Leishmania donovani* and *L. infantum* (*chagasi*), which infect a range of mammalian hosts, including humans, dogs and rodents [1]. Liver, spleen, bone marrow (BM) and lymph nodes are the major sites for parasite growth and disease pathology. Transplantation of infected kidney, liver, heart, lung, pancreas or BM has been shown to cause VL in transplant recipients, indicating lifelong parasite persistence in the viscera [2]. Moreover, malnutrition is a risk factor for the development of VL [3]. Recent experiments in protein energy-, zinc- and iron-deficient mice suggest that this effect is mediated primarily through functional failure of the lymph node barrier and increased early visceralization of the parasites [4]–[6]. Loss of the control of parasite persistence in VL causes the reactivation of parasites and relapse of the disease is frequent in the immunocompromised patients, in which the number of visceral leishmaniasis cases has been increasing recently [7]. However, the mechanisms underlying the parasite persistence in the immunocompromised condition have not been clearly clarified. To develop effective prophylactic or therapeutic strategies against VL, understanding of the precise immune mechanisms including T-cell functions in the chronic stage of infection is required [8].

The role of secondary lymphoid organs for immune responses to *Leishmania* infection has not been investigated. The *aly/aly* mouse is an autosomal recessive natural mutant C57BL/6 strain that carries a point mutation within the gene encoding NF-κB inducing kinase (NIK) [9], which prevents the induction of the non-canonical NF-κB pathway [10]. The *aly/aly* mice lack all lymph nodes and Peyer's patches with the abnormal architecture of spleen and thymus and exhibit severely impaired humoral response [9]. This mutant mouse strain has been used to examine the role of secondary lymphoid organs for immune responses to intracellular pathogens, including *Mycobacterium leprae*, *Listeria monocytogenes*, vesicular stomatitis virus, vaccinia virus, lymphocytic choriomeningitis virus and human T-cell leukemia virus [11]–[14], and different susceptibilities to these pathogens have been reported.

Organ-specific immunity has been described in various experimental VL studies in mouse models [15], [16]. The liver is the site of an acute but resolving infection. In contrast, the spleen becomes a site of parasite persistence with associated immunopathological changes [17]. In BALB/c and C57BL/6 mice, the inflammatory granuloma reaction around infected Kupffer cells is developed and the infection is resolved by 4–8 weeks after infection [18]. However, low levels of hepatic parasite persistence for 6–12 additional months occur and administration of anti-CD4 antibodies result in the relapse of hepatic quiescent *L donovani* infection [19], suggesting that CD4^+^ T cells are required for the maintenance of acquired immunity and prevention of relapse. However, no additional data explaining the underlying mechanisms of CD4^+^ T cell-mediated control of persistent parasites have been presented. Cellular and molecular interactions mediated by Kupffer cells, monocytes, CD4^+^ and CD8^+^ T cells and a number of cytokines and chemokines are required for effective hepatic granuloma formation [16]–[18], [20], [21]. Defects in these cellular and molecular factors cause ineffective parasite clearance from the liver, but most murine studies have focused on the first few weeks of infection and not the persistent stage of infection [18].

The present study is aimed to improve the understanding of mechanisms of *L. donovani* persistence in an immunocompromised condition. Our data presented herein offered a novel insight into the involvement of CD4^+^Foxp3^+^ regulatory T cells (Tregs) in *L. donovani* persistence in the liver of immunodeficient *aly/aly* mice. Moreover, treatment of infected *aly/aly* mice with anti-CD25 or anti-FR4 mAb revealed the significant reductions in both hepatic Tregs and parasite burden. These results suggest that manipulation of Tregs may provide a promising immumotherapeutic strategy for VL.

## Materials and Methods

### Mice, Parasites and Infection

Female ALY® NscJcl *aly/aly* and *aly/+* mice of 6–8 weeks of age were purchased from CLEA Japan, Inc. (Tokyo, Japan). Mice were maintained, inoculated and sacrificed within a safety facility of Hokkaido University.

A virulent line of *L. donovani* (MHOM/SU/62/2S-25M-C2) [22] was maintained by passage of the frozen stabilized parasites in NNN medium containing 5% defibrinated hemolyzed rabbit blood. Then, parasites were consecutively sub-passaged in liquid M199 medium supplemented with 15% heat-inactivated fetal calf serum (HIFCS), 25 mM HEPES and 50 µg/ml gentamycin. The stationary growth phase of subcultures with less than five passages was used for mouse inoculation. Mice were infected by injecting stationary phase promastigotes (5×10^7^) intravenously via the lateral tail vein and were sacrificed at 1, 2, 4, 8, 12, 16 and 28 weeks post infection (WPI). One group of non-infected animals was used as naïve control.

### Ethics Statement

This study was carried out under the guidance of the Institute for Laboratory Animal Research (ILAR). All animals were housed in a facility in strict accordance with the recommendations in the Guidelines for the Care and Use of Laboratory Animals of Graduate School of Veterinary Medicine, Hokkaido University, which was based on Fundamental Guidelines for Proper Conduct of Animal Experiment and Related Activities in Academic Research Institutions under the jurisdiction of the Ministry of Education, Culture, Sports, Science and Technology, Japan and approved by the American Association for Accreditation of Laboratory Animal Care (AAALAC) international. The protocol was approved by the Committee on the Ethics of Animal Experiments of Hokkaido University (Permit Number: 10-0009).

### Determination of Parasite Burdens by LDU in the Liver and qPCR in Different Tissues

Giemsa-stained impression smears of the liver were prepared and parasite burden was determined as Leishman-Donovan Units (LDU), in which LDU is the number of amastigotes per 1,000 host nuclei, multiplied by the liver weight in gram [23].

Genomic DNA (gDNA) was isolated from different tissues, including liver, spleen, BM, blood, heart, lung, kidney, brain and skin, using the QIAamp® DNA Mini Kit (Qiagen, MA, USA). Real-time quantitative (qPCR) assays were performed on the StepOne™ and the StepOnePlus™ Real-Time PCR Systems (Applied Biosystems, CA, USA), following the manufacturer's instructions. A typical 20-µl reaction mixture contained approximately 100 ng gDNA, 1× SYBR® Premix Ex Taq™ II (Takara, Tokyo, Japan), 0.4 µM each primer (Table S1) and 1× Rox™ Reference Dye. All samples were run in triplicate and underwent an initial 30 sec incubation step at 95°C, followed by 40 cycles of 5 sec at 95°C and 30 sec at 65°C for the *Leishmania surface protease gp63* gene or 60°C for the mouse *brain-derived neurotrophic factor* (*mBDNF*) gene [24], [25]. The average threshold cycle of amplification (Ct) values was determined, and standard deviation (SD) of all the reaction was analyzed by the software provided with the instrument. The relative amounts of the *gp63* gene were then calculated using standard curve method normalized to the amounts of the *mBDNF* gene.

### Histopathological Analysis of Infected Foci in the Liver

The livers were fixed in 10% neutral phosphate-buffered formalin. Paraffin-embedded organs were cut into 4 µm-thick sections, followed by staining with hematoxylin and eosin for light microscopy. For the detection of parasites, liver sections were subjected to indirect immunohistochemical staining using *L. infantum*-infected dog serum (1∶1000 dilution) [26] and horseradish peroxidase (HRP)-conjugated goat anti-dog IgG heavy and light chain antibody (1∶300; Bethyl Laboratories, TX, USA). Peroxidase was visualized using 3,3′-diaminobenzidine (DAB)-H_2_O_2_ (Wako, Tokyo, Japan) and the sections were counterstained with Mayer's hematoxylin before dehydration and mounting.

Hepatic immune responses were categorized into (1) “No granuloma”: no inflammation with no mononuclear cell (MNC) around the parasitized Kupffer cells; (2) “Immature granuloma”: less than 10 MNCs around the parasitized Kupffer cells; (3) “Mature granuloma”: epithelioid cells and more than 10 MNCs around the parasitized Kupffer cells; and (4) “Involuting granuloma”: devoid of amastigotes and tissue inflammatory nearly resolved [18], [23]. The number of infected foci with each tissue response including “No granuloma”, “Immature granuloma”, “Mature granuloma” and “Involuting granuloma” was counted for 25 consecutive microscopic fields per mouse liver at ×400 magnification.

### Determination of Foxp3-expressing T Cells by Flow Cytometry

Hepatic mononuclear cells were isolated using a 33% (vol/vol) Percoll solution, as described elsewhere [27]. Briefly, livers were minced, pressed through a stainless steel mesh and suspended in RPMI1640 medium (Sigma, MO, USA) supplemented with 3% HIFCS (wash buffer). After washing, the cells were resuspended in 33% Percoll solution containing heparin (100 U/ml) and centrifuged at 800× g for 30 min to remove liver parenchymal cells. The pellet was treated with an RBC lysis solution (155 mM NH_4_Cl, 10 mM KHCO_3_, 0.1 mM EDTA), washed and re-suspended in 2.4G2 mAb solution to block the Fc receptor before staining with antibody. Antibodies used for FACS included PE-labeled rat anti-mouse CD4 (L3T4) (BD Pharmingen, CA, USA), FITC-labeled rat anti-mouse CD8a (Lyt-2) (BD Pharmingen), APC-labeled rat anti-mouse Foxp3 (FJK-16s) (eBioscience, CA, USA) and the proper isotype staining control, according to the manufacturer's instructions. Flow cytometry analysis of the labeled cells was performed on a FACS Calibur (BD Pharmingen), running the Cell Quest program provided with the instrument. Lymphocytes were identified by forward scatter (FSC) and side scatter (SSC) characteristics, gated and further analyzed with Cell Quest software (BD Pharmingen) or FlowJo software V. 5.7.2 (Tree Star Inc., OR, USA).

### Determination of Foxp3-expressing Cells and T Cells by Immunohistochemistry

Immunohistochemical analysis of the 4 µm-thick paraffin-embedded sections of the liver was performed to determine the localization of Foxp3^+^ Tregs. After deparaffinization and rehydration, heat-induced epitope retrieval (HIER) was conducted by autoclaving at 100°C for 17 min using Target Retrieval Solution (pH 9.0) (Dako, Uppsala, Sweden). Endogenous peroxidase was blocked by incubating sections in 0.3% H_2_O_2_ in absolute methanol for 30 min at 4°C, followed by flushing with water and incubation with 10% goat serum for 1 h at room temperature (RT) to block crystallized receptor fragments. The sections were incubated overnight with rat anti-mouse/rat Foxp3 mAb, clone FJK-16s (eBioscience), in 1∶100 diluted with 0.1% Triton X in PBS (pH 7.4). For negative control sections, PBS was used instead of the primary antibody. After washing three times in PBS (5 min each), sections were incubated in 1∶100 biotin-conjugated goat anti-rat IgG (H+L) antibody (Invitrogen, MD, USA) for 30 min at RT. Sections were then washed, which was followed by incubation with streptavidin-peroxidase conjugate (Histofine SAB-PO® Kit) for 30 min at RT. The streptavidin-biotin complex was visualized with DAB-H_2_O_2_ solution, pH 7.0, for 4 min. Sections were washed in distilled water, and finally counterstained with Mayer's hematoxylin. The mean counts of Foxp3-expressing cells were assessed microscopically at 400× magnification by counting a total of 25 consecutive fields. The number of immunoreactive cells was estimated in each hepatic granuloma assembly. Values are expressed as the means of immunoreactive cells present in 25 fields.

### Double Immunofluorescence Analysis

Double immunofluorescence staining was also conducted to locate Tregs in the liver. Formalin-fixed and paraffin-embedded liver sections were subjected to the deparaffinization, rehydration and HIER as described above. After blocking of crystallized receptor fragments with 10% goat serum, sections were incubated overnight with rat anti-mouse/rat Foxp3 mAb (clone FJK-16s; 1∶100; eBioscience) at 4°C. Then, the sections were incubated with FITC-goat anti rat IgG (1∶200; Zymed, CA, USA) for 30 min at RT and successively incubated in 10% donkey or rabbit serum to block the crystallized receptor fragments. For double staining of Foxp3-expressing cells and T cells, the sections were incubated with rabbit anti-mouse CD3 mAb (1∶200; Nichirei) overnight at 4°C and then with TRITC-donkey anti rabbit IgG (1∶200; Abcam, MA, USA) for 30 min at RT. On the other hand, double staining of Foxp3-expressing cells and *L. donovani* amastigotes was conducted using *L. infantum*-infected dog serum (1∶1,000) and TRITC-goat anti dog IgG (1∶200; Rockland, PA, USA). Finally, the sections were mounted using a Fluoromount™ (DBS, CA, USA) and examined under an IX70 confocal microscope (Olympus, Tokyo, Japan).

### Laser Microdissection, RNA Purification and qRT-PCR

Laser microdissection (LMD) was performed in RNase-free conditions as described previously [28]. Cryosections of 7 µm thickness were prepared from the frozen livers of naïve and infected mice and embedded in Tissue-Tek OTC compound (Sakura, Tokyo. Japan). The sections were mounted on glass slides pre-coated with LMD films (Meiwafosis, Osaka, Japan) and fixed with absolute methanol for 3 min at 4°C. After staining with 0.5% toluidine blue for 10 sec, approximately 20 “Mature granulomas” were microdissected from each frozen liver sample by using Ls-Pro300 (Meiwafosis).

Total RNA was purified from the frozen whole liver tissue and microdissected “Mature granulomas”, using the RNAqueous®-Micro Kit (Ambion, Texas, USA). Expression levels of *Foxp3*, *TGF-β* and *IL-10* mRNA were determined by quantitative RT-PCR (qRT-PCR) using the PrimeScript™ RT Reagent Kit (Takara) and the relative number of these molecules to 1000 housekeeping *glyceraldehyde-3-phosphate dehydrogenase* (*GAPDH*) was calculated using a standard curve method. The PCR reaction was performed as described above using primers shown in Table S1 [29], [30].

### Inhibition of CD4^+^ Foxp3^+^ Treg Function

At 26 WPI, three *L. donovani*-infected *aly/aly* mice were intraperitoneally injected three times every other day with 0.5 mg of rat anti-mouse CD25 mAb (clone PC61; Biolegend, CA, USA), 0.05 mg of rat anti-mouse FR4 mAb (clone TH6; Biolegend) or 0.5 mg of rat IgG (Jackson ImmunoResearch, PA, USA) as a control. The mice were euthanized at 10 days post-antibody injection for examination of host responses as described above.

### Statistics

Statistical differences between *aly/aly* mice and *aly/+* mice at the indicated time points were tested using Student's *t*-test (Microsoft Excel software) and two-way ANOVA as well as post hoc Bonferroni test (Prism software version 5, GraphPad, CA, USA). All data are presented as the mean values ± SE unless otherwise stated. *p*<0.05 was considered as statistically significant.

## Results

### Long-term Persistence of *L. donovani* in the Liver of *aly/aly* Mice

**Figure 1:**
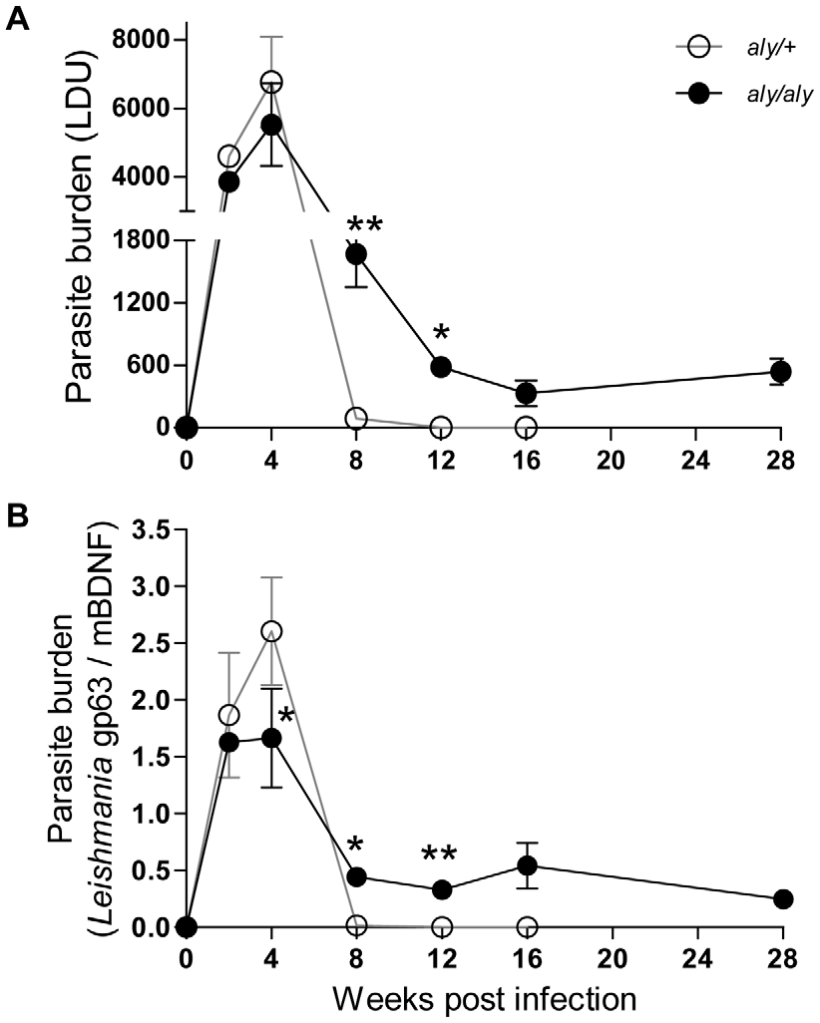
Long-term persistence of *L. donovani* amastigotes in the liver of *aly/aly* mice. Promastigotes of *L. donovani* were intravenously inoculated into *aly/+* (○) and *aly/aly* (•) mice, and at the indicated time points, parasite burdens in the liver were determined as LDU (A), and relative amounts of *Leishmania* gp63 gene to mouse housekeeping BDNF gene by qPCR (B). A typical result of two individual experiments is shown. Data are the mean ± SE for three mice of each strain. * *p*<0.05; ** *p*<0.01.

Long-term persistence after clinical cure of the primary infection is a characteristic feature of many intracellular pathogens, including protozoan parasites of the genus *Leishmania*, but the underlying mechanisms are not fully understood [31]. We measured parasite burdens in the livers of *aly/+* and *aly/aly* mice for up to 28 WPI by two different methods. The number of amastigotes in hepatic impression smears was expressed as LDU (Figure 1A), and relative amounts of *Leishmania gp63* gene to *mBDNF* gene were determined by qPCR (Figure 1B). In *aly/+* mice, parasite burden peaked at 4 WPI and reduced to near-baseline levels by 8 WPI. In *aly/aly* mice, parasite burden also peaked at 4 WPI but the maximum parasite burden was lower than that of *aly/+* mice. Although the parasite load decreased by 8 WPI as observed in *aly/+* mice, the parasite persisted in the liver of *aly/aly* mice during the observation period of 28 WPI (Figure 1). Persistent *L. donovani* infection was also demonstrated in the spleen and BM of both mice strains but the parasite burden was much higher in *aly/aly* mice during the chronic phase of infection. Nevertheless, parasite was not detected in the skin and internal organs, such as lung, kidney, heart and brain during infection by qPCR (data not shown).

### Delayed and Impaired Hepatic Granuloma Maturation in *aly/aly* Mice During *L. donovani* Infection

Efficient granuloma development around infected Kupffer cells is a key event in the control of hepatic *L. donovani* infection [8], [16], [18]. The infected foci in the liver were examined and made a quantitative analysis of granuloma formation around the parasitized Kupffer cells. The progression of granuloma formation from “No granuloma” to “Immature granuloma”, “Mature granuloma” and finally “Involuting granuloma” was observed in *aly/aly* mice as well as *aly/+* mice (Figure S1), indicating that *aly/aly* mice have ability to generate hepatic cell-mediated immunity to some extent as shown in the previous study [32].

**Figure 2:**
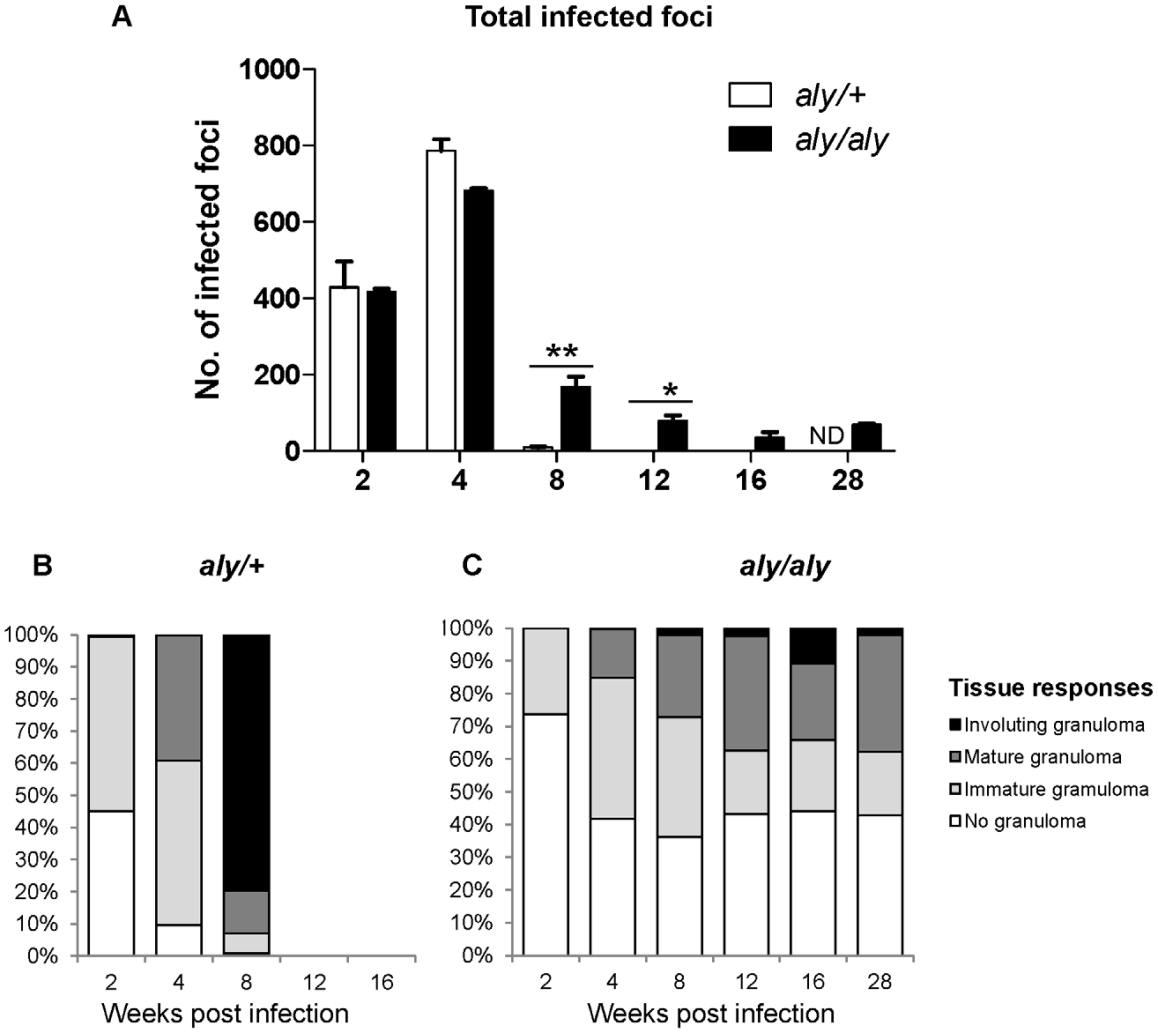
Delayed and impaired hepatic granuloma maturation in *aly/aly* mice during *L. donovani* infection. Host immune responses to each infected focus were quantitatively analyzed in *aly/+* (open bars) and *aly/aly* (filled bars) mice during the course of *L. donovani* infection. Total number of infected foci was counted from 25 consecutive microscopic fields (A). The proportion of “No granuloma”, “Immature granulomas”, “Mature granulomas” and “Involuting granuloma” of *aly/+* (B) and *aly/aly* (C) mice are estimated. A typical result of two individual experiments is shown. Data are the mean ± SE for three mice of each strain. ND, not determined; * *p*<0.05; ** *p*<0.01.

The number of infected foci was well correlated with the hepatic parasite loads (Figure 1); the number of foci in *aly/+* mice reached a peak at 4 WPI and was drastically reduced at 8 WPI (Figure 2A) and the involuting granuloma was well formed (Figure 2B). However, the number of involuting granuloma in the liver of *aly/aly* mice were much less than those in *aly/+* mice at 4 and 8 WPI (Figure 2C) while the 30–40% of the infection foci with no granulomas was found in the liver of *aly/aly* mice at 4–16 WPI. These may reflect that effective but insufficient clearance of the parasites in granuloma of *aly/aly* livers renders persistent release of the parasites, which results in increased proportion of infected Kupffer cells in the later stages.

### Expansion of CD4^+^Foxp3^+^ Tregs in the Liver of *aly/aly* Mice During Persistent *L. donovani* Infection

Foxp3^+^ Tregs influence immunity to viral, bacterial or parasitic infections [33]. To begin to characterize the mechanism by which parasites persist in the liver, we examined whether Tregs expand in the livers of *aly/aly* mice and where they localize during *L. donovani* infection.

**Figure 3:**
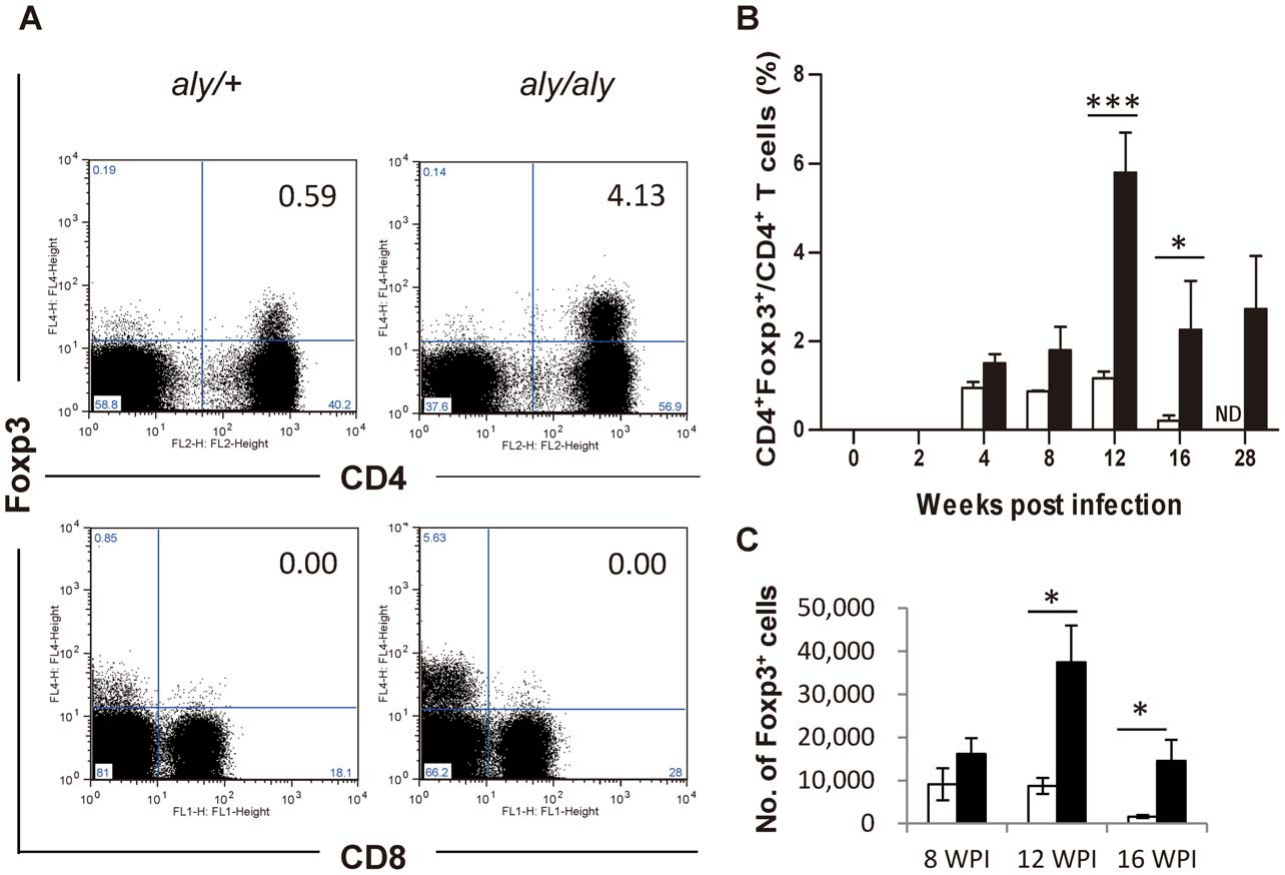
Expansion of CD4^+^Foxp3^+^ Tregs in the liver of *aly/aly* mice during persistent *L. donovani* infection. Flow cytometry analysis of CD4^+^Foxp3^+^ and CD8^+^Foxp3^+^ Tregs among the hepatic lymphocytes. (A) Representative data of the gated lymphocytes extracted from the liver at 12 WPI of *aly/+* (left) and *aly/aly* mice (right), stained with anti-Foxp3 and anti-CD4 or anti-CD8 mAb. (B) The proportion of CD4^+^Foxp3^+^ T cells to CD4^+^ T cells in *aly/+* (open bars) and *aly/aly* (filled bars) mice. (C) The total number of CD4^+^Foxp3^+^ T cells in *aly/+* (open bars) and *aly/aly* (filled bars) mice at 8–16 WPI. A typical result of two individual experiments is shown. Data are the mean ± SE for three mice of each strain. ND, not determined; * *p*<0.05; *** *p*<0.001.

Flow cytometry analysis of hepatic lymphocytes revealed no expansion of CD8^+^Foxp3^+^ T cells in the liver during *L. donovani* infection in either strain of mice (Figure 3A). In contrast, CD4^+^Foxp3^+^ T cells were first detected at 4 WPI in both strains of mice. In *aly/aly* mice, the proportions of CD4^+^Foxp3^+^ T cells to CD4^+^ T cells (Figure. 3B) as well as the absolute number of CD4^+^Foxp3^+^ T cells (Figure 3C) were higher than those of *aly/+*mice especially at 8–16 WPI although the total number of hepatic CD4^+^ T cells was not significantly different between *aly/+* and *aly/aly* mice (Figure S2).

### Presence of Foxp3^+^ Tregs Inside Granulomas and Perivascular Neo-lymphoid Areas in the *L. donovani*-infected liver of *aly/aly* Mice

**Figure 4:**
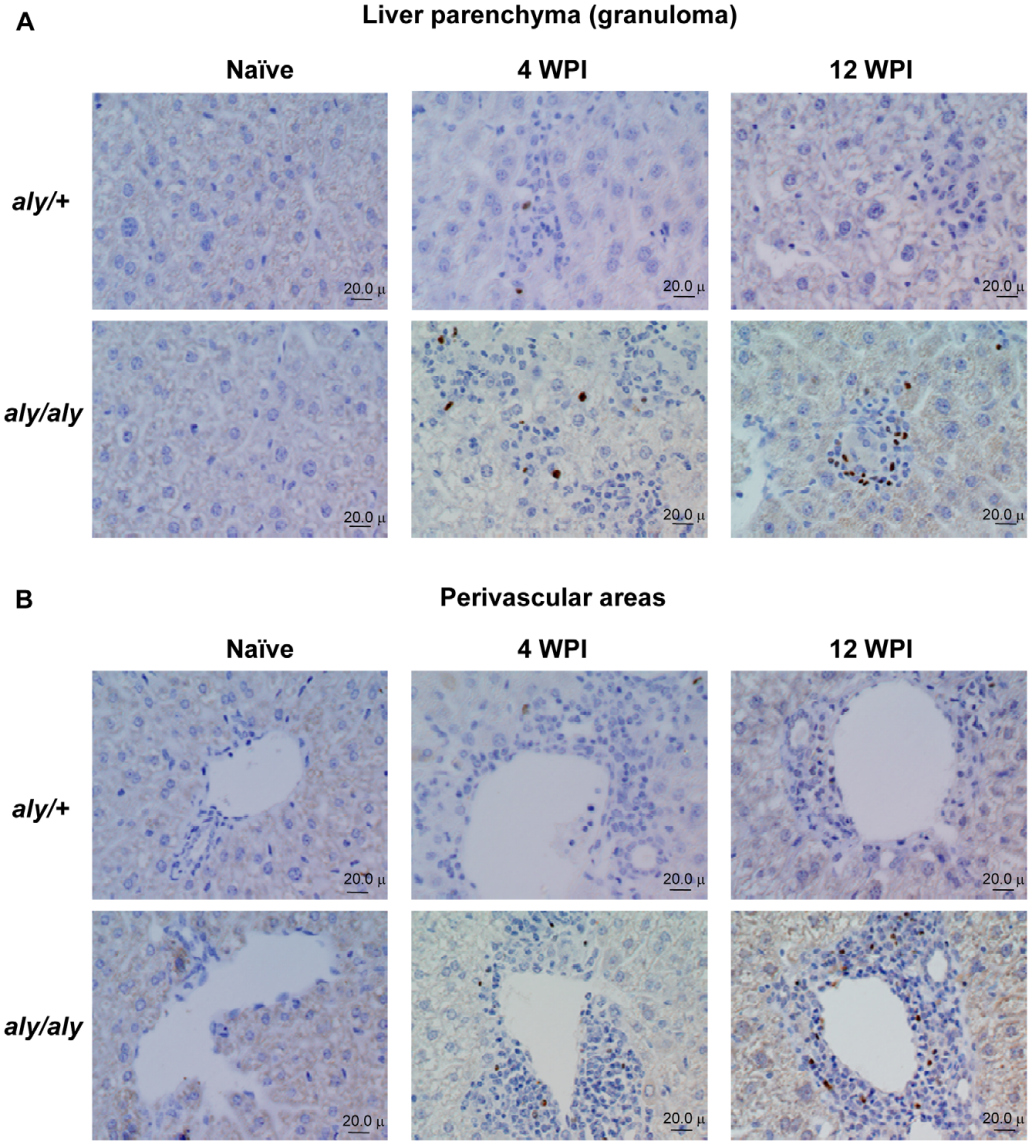
Presence of Foxp3^+^ Tregs inside granulomas in the liver of *L. donovani*-infected *aly/aly* mice. Localization of Foxp3-expressing cells in liver sections from *aly/+* mice and *aly/aly* mice during the course of *L. donovani* infection was analyzed by immunohistochemistry using anti-Foxp3 mAb. Typical reactions in the liver parenchyma including “Immature granuloma” and “Mature granulomas” (A) and perivascular areas (B) in uninfected naïve mice and infected mice at 4 and 12 WPI are shown. The brown pigments represent Foxp3-immunoreactive cells.

There have been no reports describing the localization of Foxp3-expressing cells in the liver during VL. To address this, we stained Foxp3 in liver sections of naïve and *L. donovani*-infected *aly/+* and *aly/aly* mice. Foxp3-expressing cells were localized in the “Immature granuloma” and “Mature granulomas” as well as the perivascular areas of infected *aly/aly* mice. Furthermore, the density of Tregs increased, especially in the perivascular areas, during the course of infection (Figure 4A and B). Development of such abnormal lymphocyte infiltration or neo-lymphoid aggregates at perivascular areas is a feature found in *aly/aly* and other alymphoplastic mice [32]. In addition, the frequency of “Mature granulomas” containing more than 5 Tregs increased during infection in *aly/aly* mice (5% at 4 WPI, 18% at 12 WPI and 39% at 28 WPI), suggesting the accumulation of Tregs at sites of inflammatory foci. On the other hand, Foxp3-positive Tregs were limited to the parenchyma, granulomas and perivascular areas at 4 WPI and hardly detectable in the liver of infected *aly/+* mice at 12 WPI (Figure 4A and B).

**Figure 5:**
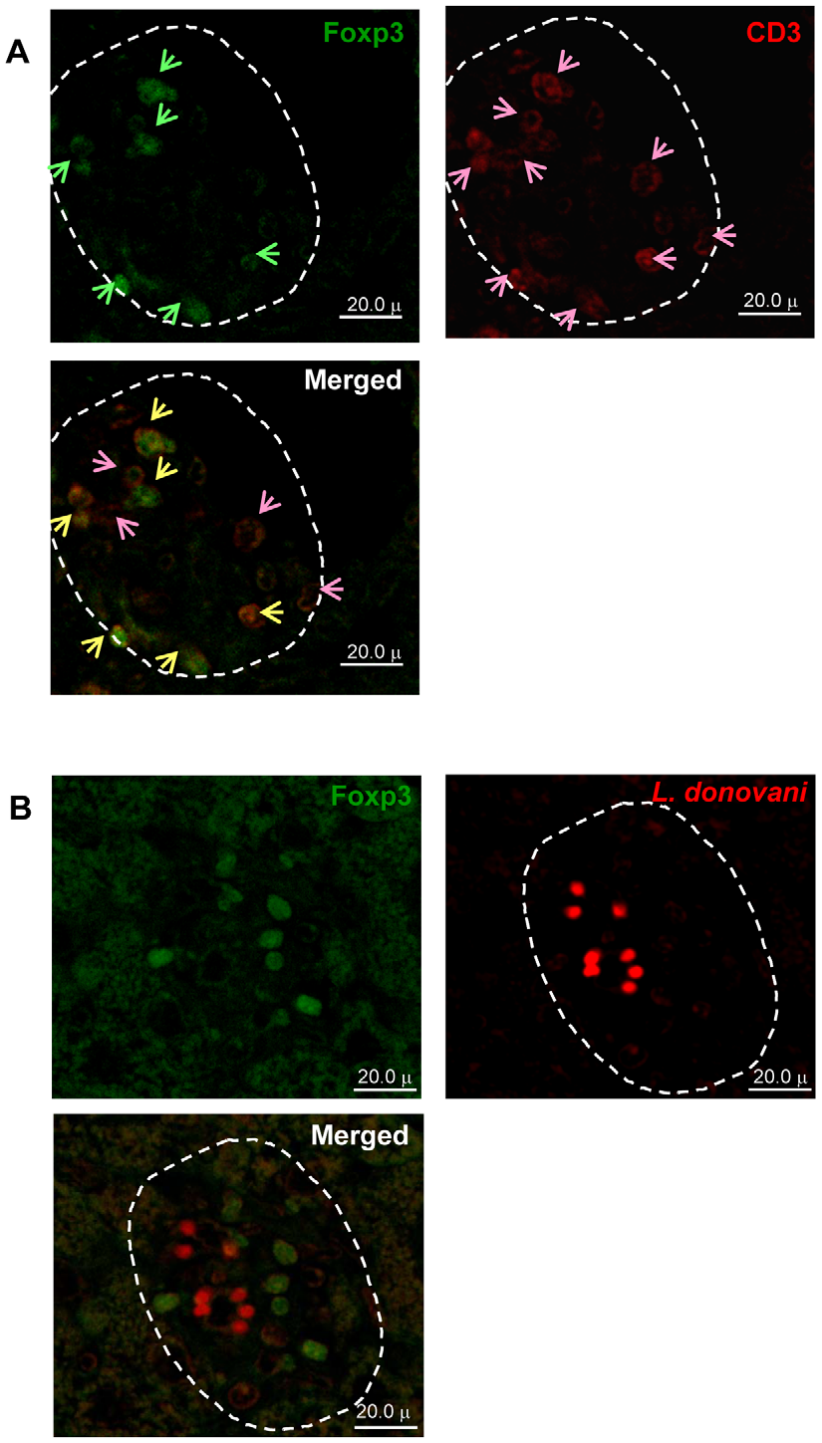
Localization of Foxp3-expressing cells in *L. donovani*-infected granuloma of *aly/aly* mice. Representative “Mature granuloma” in the liver sections of *aly/aly* mice at 12 WPI using single and double immunostaining. (A) Foxp3^+^ cells (green; upper left), CD3^+^cells (red; upper right) and a merged image (lower) showed CD3^+^Foxp3^+^ (yellow arrows) and CD3^+^Foxp3^−^ (pink arrows) cells. (B) Foxp3^+^ cells (green; upper left), *Leishmania* amastigotes (red; upper right) and a merged image (lower). Arrows indicated the positive stained cells.

Double immunofluorescence analysis of hepatic granuloma revealed that Foxp3-expressing cells (green in Figure 5A) and CD3^+^ cells (red in Figure 5A) were present in the granuloma, and Foxp3^+^ cells expressed CD3 molecules (Figure 5A-merged image). Some CD3^+^Foxp3^+^ cells (yellow arrows in Figure 5A-merged image) were adjacent to the CD3^+^Foxp3^−^ cells (pink arrows in Figure 5A-merged image). In addition, *L. donovani* amastigotes (red in Figure 5B) were surrounded by Foxp3^+^ cells (green in Figure 5B) in the hepatic granuloma. These results suggested that the interaction among parasitized cells (Kupffer cells), CD3^+^Foxp3^+^ cell (Tregs) and CD3^+^Foxp3^−^ cells (non Tregs, probably CD4^+^ and/or CD8^+^ effector T cells).

### Association of *Foxp3* and *IL-10* mRNA Expression in “Mature Granulomas”

**Figure 6:**
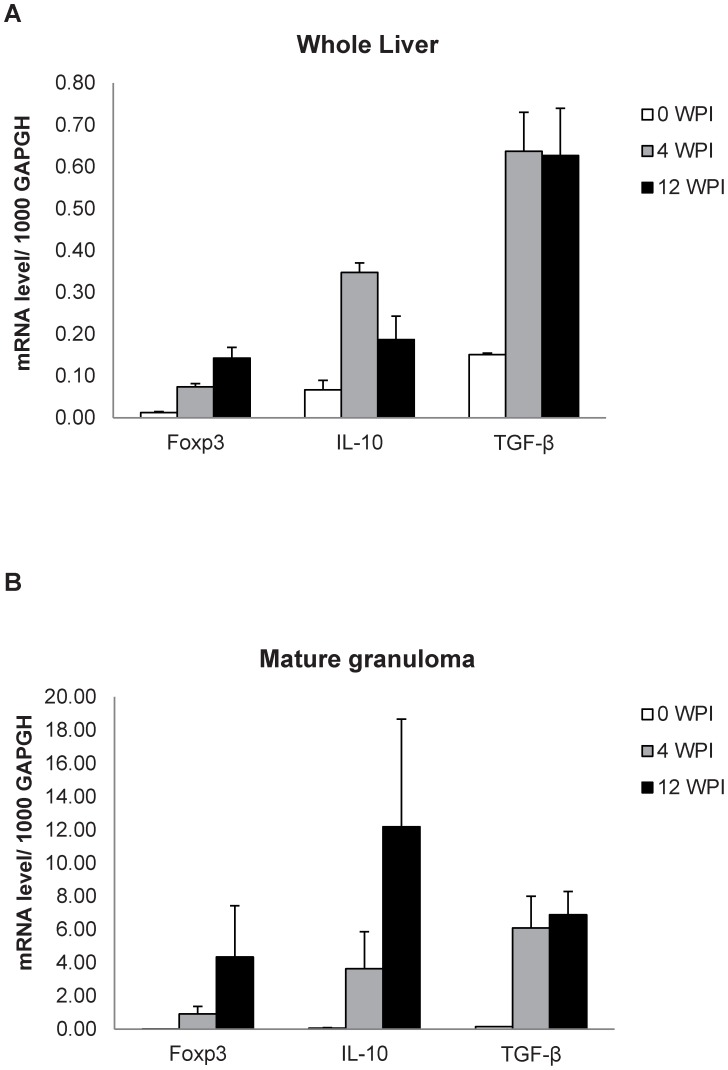
Expression of *Foxp3*, *TGF-β* and *IL-10* mRNAs in the liver of *L. donovani*-infected *aly/aly* mice. Relative mRNA levels of *Foxp3*, *IL-10* and *TGF-β* to 1000 *GAPDH* were estimated in the whole liver samples (A) and microdissected hepatic parenchymal or granuloma tissue samples (B) of naïve (0 WPI) and *L. donovani* infected *aly/aly* mice at 4 and 12 WPI. A typical result of two individual experiments is shown. Data are the mean ± SE for three mice at each indicating time point.

Evidence has accumulated regarding the essential roles of Tregs in the control of a variety of physiological and pathological immune responses, but it is still obscure how Tregs control other lymphocytes at the molecular level [34]. Quantitative RT-PCR was performed for *Foxp3*, *IL-10* and *TGF-β* mRNA levels in the whole liver and micro-dissected “Mature granulomas” liver tissue samples of *L. donovani*-infected *aly/aly* mice. The *Foxp3* mRNA expression was increased after infection in the whole liver (Figure 6A) and mature granuloma samples (Figure 6B). Although the *TGF-β* mRNA transcripts showed similar levels at 4 and 12 WPI in both tissue samples, the levels of *IL-10* mRNA markedly increased in mature granuloma but not whole liver samples at 12 WPI (Figure 6A and B), suggesting that IL-10 may be involved in function of Tregs.

### Enhanced Effector Immune Responses and Declined Parasite Burden in the Liver After Inhibition of Treg Function in *L. donovani*-infected *aly/aly* Mice

**Figure 7:**
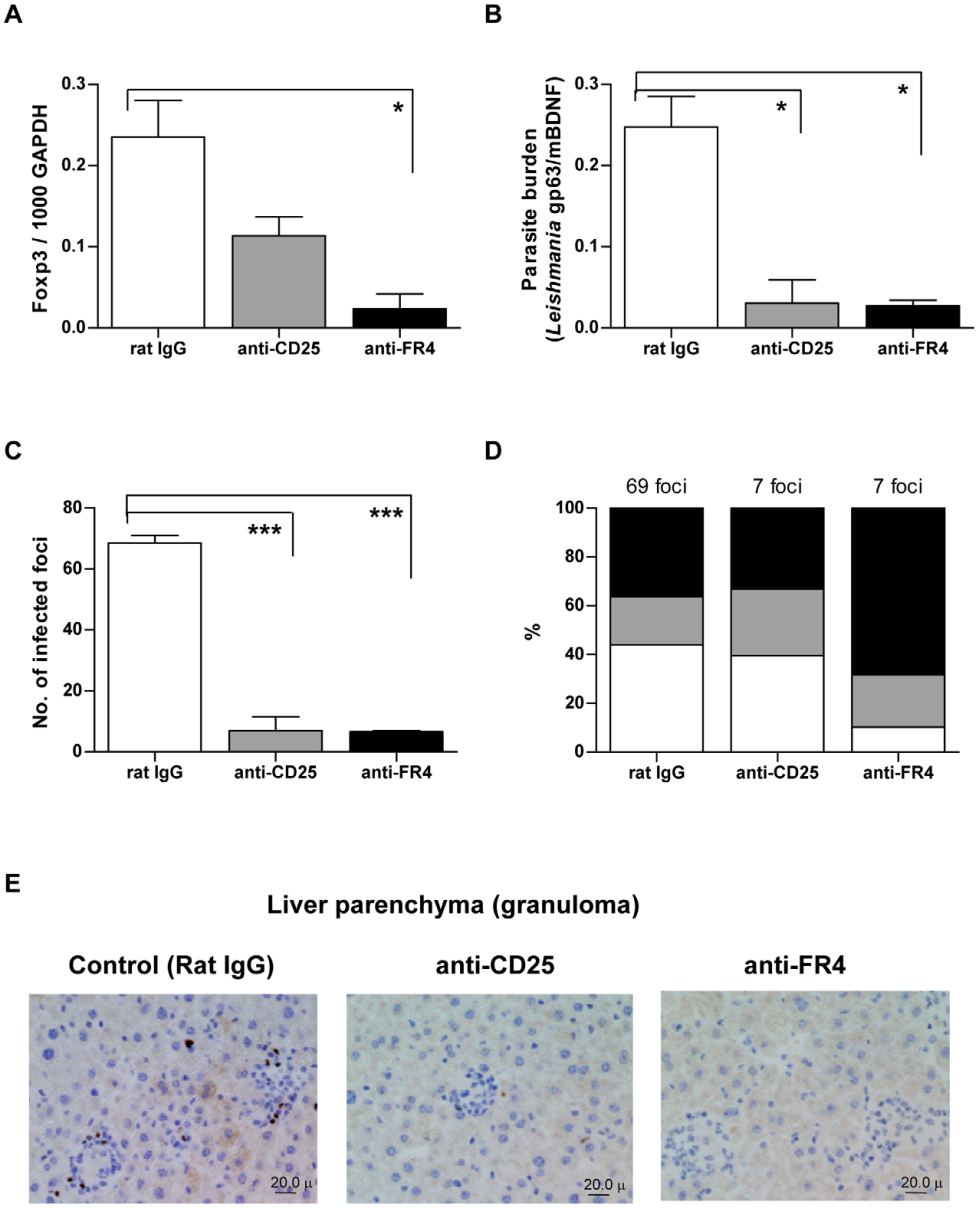
Effects of anti-Treg antibodies on Foxp3 expression, parasite burden and infected foci in the liver. The *L. donovani*-infected *aly/aly* mice were intraperitoneally injected with anti-CD25 or anti-FR4 mAb at 26 WPI and hepatic immune responses were examined after 10 days of injection. (A) *Foxp3* mRNA expression by RT-PCR. (B) Parasite burden expressed by qPCR. (C) The number of infected foci from 25 consecutive microscopic fields. (D) Frequency of mature (black column) and immature (grey column) granuloma, and no response (white column) in the infected foci. (E) Localization of Foxp3-stained cells in the liver parenchyma including “Immature granulomas” and “Mature granulomas”. The brown pigments represent Foxp3-immunoreactive cells. * *p*<0.05; *** *p*<0.001.

Manipulation of Tregs by treatment with antibodies has been used to examine the roles of Tregs in many infectious diseases [33]. Effects of anti-CD25 and anti-FR4 mAb on hepatic immune responses in *L. donovani*-infected *aly/aly* mice at 26 WPI were examined. Ten days after injection with anti-CD25 or anti-FR4, reduction in *Foxp3* mRNA expression was observed (Figure 7A). This reduced *Foxp3* mRNA expression was associated with decreases in parasite burden (Figure 7B) and infected foci (Figure 7C). Instead, the frequency of “Mature granulomas” was increased after treatment with especially anti-FR4 mAb (Figure 7D), suggesting that depletion of Tregs can activate hepatic cellular immune responses and accelerate parasite killing. Furthermore, immunohistochemical analysis confirmed a reduction in Foxp3-immunoreactive cells in the liver parenchyma, granulomas (Figure 7E) and perivascular neo-lymphoid areas (data not shown).

## Discussion

In the present study, *aly/aly* mice were used as an immunodeficient VL murine model and immunohistopathologically investigated during *L. donovani* infection for up to 28 WPI. CD4^+^Foxp3^+^ T cells were increased in the granulomas and perivascular areas of the liver in the chronic phase and the impairment of granuloma maturation was observed. The depletion of Tregs by the administration of either anti-CD25 or anti-FR4 mAb resulted in significant reductions in hepatic Tregs, infected foci and parasite burden. To our knowledge, this is the first definitive evidence that CD4^+^Foxp3^+^ Tregs are involved in hepatic *L. donovani* persistence in a murine model of VL.

The *aly/aly* mice have been used to examine the role of secondary lymphoid organs on immune responses in various infection models. Disruptive architecture of the thymus and spleen could affect the development and expansion of T cells. Several studies using bone marrow chimeras between *aly/aly* and wild type mice showed that antiviral CTL responses were clearly improved in the wild type environment [11]. However, expansion of CD25^+^CD4^+^ Treg is impaired in the spleen of *aly/aly* mice [35]. This suggests that expansion of functional CD4^+^Foxp3^+^ Treg in the liver of *aly/aly* mice during *L. donovani* infection is likely related to the parasite persistence but not to the structural defects of secondary lymphoid organs although this possibility will be confirmed by BM chimera experiments in future.

The NIK gene mutation may contribute to other immune defects due to the partial blocked NF-κB activation [10], [36]. NF-κBp52 knockout mice showed less parasite burden in the liver, perhaps due to less number of B cells (unpublished data; Ato M., Kaye PM). NF-κBp50 (NF-κB1) would be important for TNF/TLR signaling which is involved in canonical TLR/TNFR signaling for activation of dendritic cells and macrophages. NIK is associated in CD40/LT-αβR but not in TNF. CD40 signaling is one of DC activation factors, but the function of DC of *aly/aly* are controversial. Yamada et al [36] has reported that DC from *aly/aly* mice exhibit grossly normal development and function. However, Tamura et al [35] had reported that DCs from *aly/aly* mice showed impaired antigen presentation ability. Lower hepatic parasite loads was unexpectedly observed in *aly/aly* mice than *aly/+* mice in the first 4 WPI. This may be not due to lower number of the sessile Kupffer cells (unpublished data), but associated with the strong innate immunity as reported during *Listeria monocytogenes* infection in *aly/aly* mice [12]. Partial hepatic granuloma progression and neo-lymphoid aggregates in *aly/aly* mice imply that mice lacking secondary lymphoid tissues can still generate T cell-mediated immune responses to some extent [32].

Although anti-CD25 mAbs have been used for depletion of Tregs in various experimental cases, administration of anti-FR4 mAb also reduced Treg numbers and provoked effective tumor immunity [37]–[39]. In the present study, 10 days after the third injection of infected *aly/aly* mice with anti-CD25 and anti-FR4 mAb, the hepatic parasite burdens were reduced by 88% and 89% of that of control mice, respectively (Figure 7B). Likewise, treatment with either anti-CD25 or anti-FR4 mAb also reduced parasite burdens in the spleen and BM (Figure S3). The reason why anti-FR4 mAb was more effective than anti-CD25 mAb in reducing parasite burden is unknown, but the present study is the first to report effectiveness of anti-FR4 mAb to control systemic infection of *L. donovani* in mice. Thus, anti-FR4 antibodies may be an alternative measure to manipulate Tregs in chronic VL. However, since anti-CD25 mAb can also affect effector T cells and effective immunity [39], and anti-FR4 mAb can also deplete a small population of CD4^+^ Foxp3^−^ T cells in the lymph node [38], probably including IL-10-producing conventional CD4^+^ T cells, further studies of the role of Tregs in VL are required.

Studies of Tregs in cutaneous leishmaniasis demonstrated the involvement of CD4^+^CD25^+^ Tregs in cutaneous leishmaniasis caused by *L. major* [40], [41] and *L. amazonensis* in mice [42] and by *L. braziliensis* in humans [43]. Regarding VL, the role of Tregs is uncertain and the primary source of IL-10 is controversial. In the spleen of VL patients in India, CD4^+^CD25^−^Foxp3^−^ cells were identified as the major producers of IL-10 [44]. In *L. infantum*-infected BALB/c mice, CD4^+^CD25^+^ Foxp3^+^ cells expanded in a pooled fraction of draining lymph nodes and spleen cells at 7 and 28 days of infection [45]. In *L. donovani*-infected BALB/c mice, the number of splenic CD4^+^ CD127^dim^CD25^+^GITR^+^ T cells expressing higher Foxp3 and IL-10 increased at 21 days of infection [46]. IL-10 production by splenic CD4^+^CD25^−^Foxp3^−^ IL10^+^ T cells, representing type 1 regulatory T (Tr1) cells, was a strong correlate of disease progression in *L. donovani*-infected C57BL/6 mice [47]. Further analyses using quantitative RT-PCR of *IL-10* and *Foxp3* transcripts in selected populations of CD25^+^ and CD25^−^ enriched hepatic CD4^+^ T cells, and/or by intracellular cytokine staining, will elucidate the issue. Nevertheless, in the present study, Treg and IL-10 augment immunosuppressive effects in hepatic granuloma of *L. donovani*-infected *aly/aly* mice. Maintenance of relatively higher expression levels of *TGF-β* in the chronic phase of the infection in *aly/aly* mice may be related to the generation and maintenance of CD4^+^Foxp3^+^ Tregs [48] rather than the inhibition of granuloma maturation [49].

In conclusion, we focused on immune responses to the chronic phase of murine VL caused by *L. donovani* infection in an immunodeficient host. In the last decade when the number of visceral leishmaniasis in immunocompromised patients has been increasing, our data presented herein offered a novel insight into the possibly involvement of CD4^+^Foxp3^+^ Tregs in persistent *L. donovani* infection in the liver of immunodeficient hosts. The manipulation of Tregs may provide a promising immumotherapeutic strategy for VL.

## Supporting Information

Figure S1 **Hepatic immune response and granuloma formation in** ***aly/+*** **and** ***aly/aly*** **mice 2–8 weeks after** ***L. donovani*** **infection.** Representative hepatic immune responses to infected foci in liver sections by staining with HE and immunostaining with anti-*Leishmania* serum for *aly/+* (A–D and E–H, respectively) and *aly/aly* (I–L and M–P, respectively) mice are shown. Immune responses to parasitized Kupffer cells were categorized into four types; “No granuloma” (A and E, I and M), “Immature granuloma” (B and F, J and N), “Mature granuloma” (C and G, K and O) and “Involuting granuloma” (D and H, L and P). The brown pigments indicate *L. donovani* amastigotes. The yellow circles indicate *L. donovani*-infected foci in each type of tissue responses.
(PDF)

Figure S2 **Proportion of CD4^+^Foxp3^+^ and CD4^+^ T cells to gated hepatic lymphocytes during the course of** ***L. donovani*** **infection by flow cytometry analysis.** Hepatic lymphocytes were isolated from the liver of *aly/+* and *aly/aly* mice at indicated time points after *L. donovani* infection and stained with anti-CD4, CD8 and Foxp3 antibodies. Percentage of CD4^+^ T cells (white bars) and CD4^+^Foxp3^+^ T cells (black bars) of *aly/+* (A) and *aly/aly* mice (B) are shown.
(PDF)

Figure S3 **Effects of anti-Treg antibody treatment on parasite burden in the spleen and bone marrow.** The *L. donovani*-infected *aly/aly* mice were intraperitoneally injected with anti-CD25 or anti-FR4 mAb at 26 WPI, and parasite burden in the spleen (A) and bone marrow (B) was estimated by qPCR after 10 days of antibody treatment. * *p*<0.05; ** *p*<0.01.
(PDF)

Table S1 **Target genes and primers for qPCR and RT-PCR used in this study.**
(DOCX)
