# Supplementary material for: Involvement of CD4+ Foxp3+ Regulatory T Cells in Persistence of Leishmania donovani in the Liver of Alymphoplastic aly/aly Mice
Source: PLoS Negl Trop Dis. 2012 Aug 21;6(8):e1798. doi: 10.1371/journal.pntd.0001798 (PMC3424244; doi:10.1371/journal.pntd.0001798)
Supplement: Figure S1 — Hepatic immune response and granuloma formation in aly/+ and aly/aly mice 2–8 weeks after L. donovani infection. Representative hepatic immune responses to infected foci in liver sections by staining with HE and immunostaining with anti-Leishmania serum for aly/+ (A–D and E–H, respectively) and aly/aly (I–L and M–P, respectively) mice are shown. Immune responses to parasitized Kupffer cells were categorized into four types; “No granuloma” (A and E, I and M), “Immature granuloma” (B and F, J and N), “Mature granuloma” (C and G, K and O) and “Involuting granuloma” (D and H, L and P). The brown pigments indicate L. donovani amastigotes. The yellow circles indicate L. donovani-infected foci in each type of tissue responses. (PDF) [file pntd.0001798.s001.pdf]

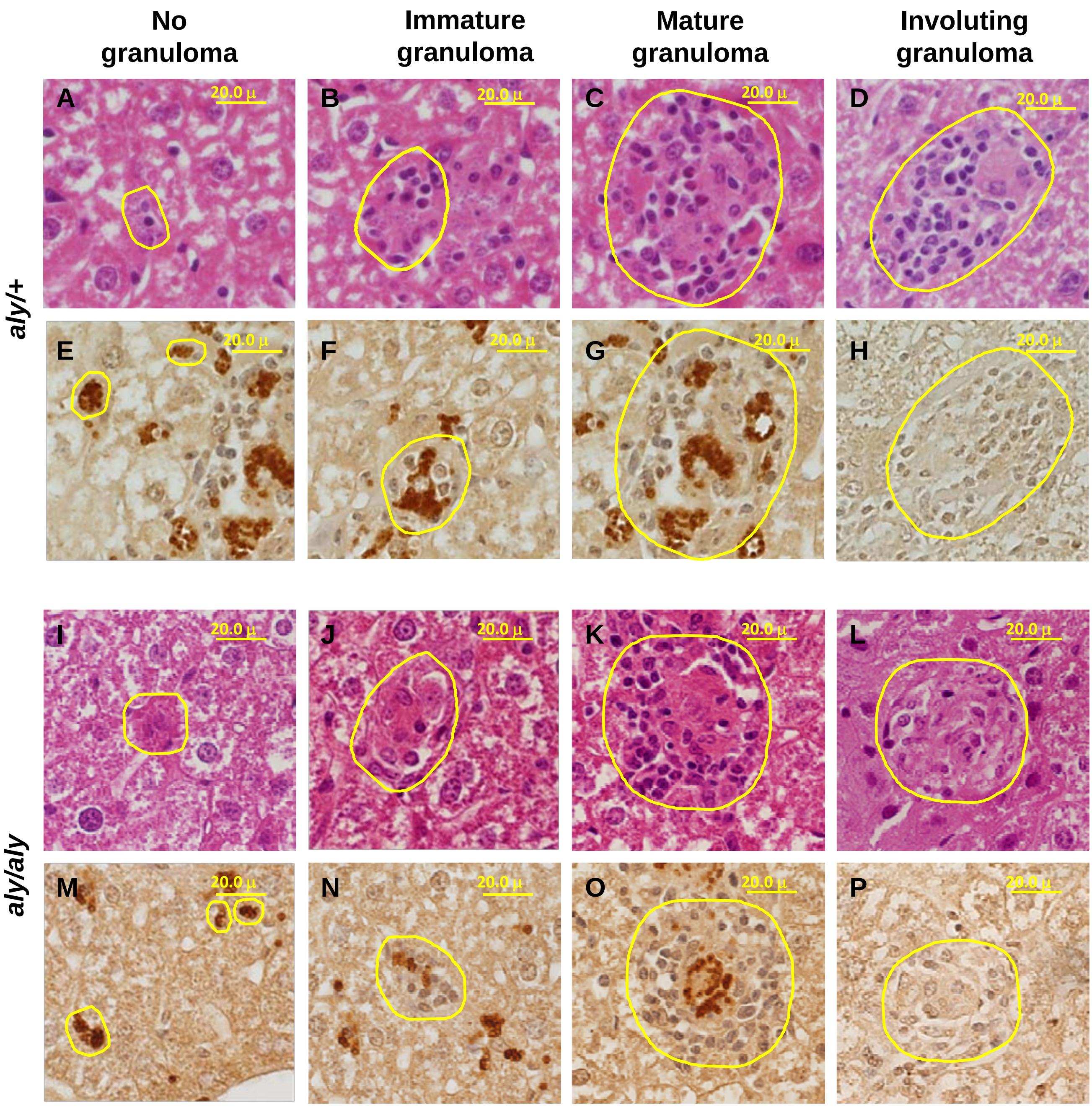

Figure S1. **Hepatic immune response and granuloma formation in *aly*/+ and *aly/aly* mice 2-8 weeks after *L. donovani* infection.**

Representative hepatic immune responses to infected foci in liver sections by staining with HE and immunostaining with anti-*Leishmania* serum for *aly*/+ (A-D and E-H, respectively) and *aly/aly* (I-L and M-P, respectively) mice are shown. Immune responses to parasitized Kupffer cells were categorized into four types; “No granuloma” (A and E, I and M), “Immature granuloma” (B and F, J and N), “Mature granuloma” (C and G, K and O) and “Involuting granuloma” (D and H, L and P). The brown pigments indicate *L. donovani* amastigotes. The yellow circles indicate *L. donovani*-infected foci in each type of tissue responses.
