# Supplementary material for: Involvement of CD4+ Foxp3+ Regulatory T Cells in Persistence of Leishmania donovani in the Liver of Alymphoplastic aly/aly Mice
Source: PLoS Negl Trop Dis. 2012 Aug 21;6(8):e1798. doi: 10.1371/journal.pntd.0001798 (PMC3424244; doi:10.1371/journal.pntd.0001798)
Supplement: Figure S2 — Proportion of CD4+Foxp3+ and CD4+ T cells to gated hepatic lymphocytes during the course of L. donovani infection by flow cytometry analysis. Hepatic lymphocytes were isolated from the liver of aly/+ and aly/aly mice at indicated time points after L. donovani infection and stained with anti-CD4, CD8 and Foxp3 antibodies. Percentage of CD4+ T cells (white bars) and CD4+Foxp3+ T cells (black bars) of aly/+ (A) and aly/aly mice (B) are shown. (PDF) [file pntd.0001798.s002.pdf]

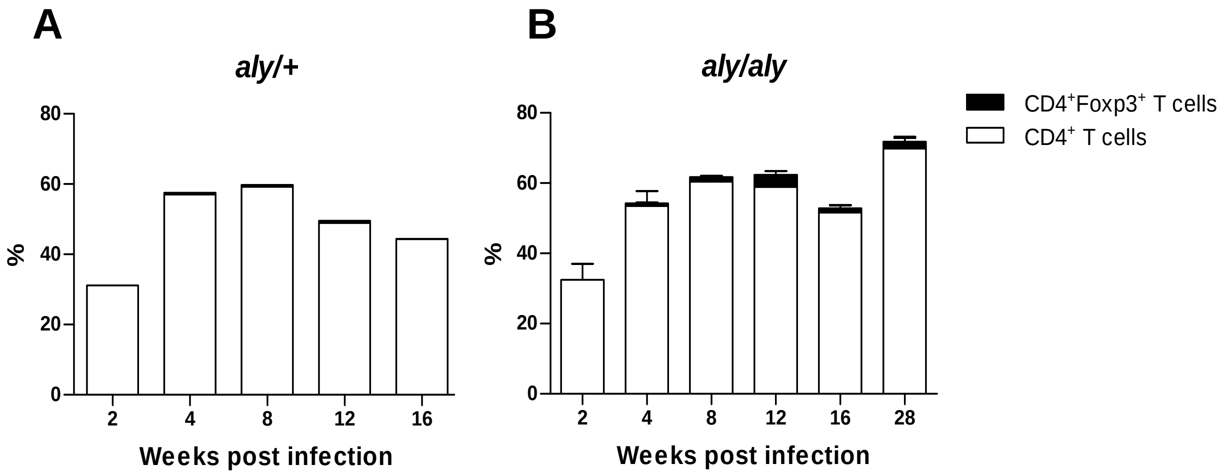

Figure S2. **Proportion of CD4$^+$Foxp3$^+$ and CD4$^+$ T cells to gated hepatic lymphocytes during the course of *L. donovani* infection by flow cytometry analysis.**
Hepatic lymphocytes were isolated from the liver of *aly/*+ and *aly/aly* mice at indicated time points after *L. donovani* infection and stained with anti-CD4, CD8 and Foxp3 antibodies. Percentage of CD4$^+$ T cells (white bars) and CD4$^+$Foxp3$^+$ T cells (black bars) of *aly/*+ (A) and *aly/aly* mice (B) are shown.
