# Supplementary material for: Involvement of CD4+ Foxp3+ Regulatory T Cells in Persistence of Leishmania donovani in the Liver of Alymphoplastic aly/aly Mice
Source: PLoS Negl Trop Dis. 2012 Aug 21;6(8):e1798. doi: 10.1371/journal.pntd.0001798 (PMC3424244; doi:10.1371/journal.pntd.0001798)
Supplement: Figure S3 — Effects of anti-Treg antibody treatment on parasite burden in the spleen and bone marrow. The L. donovani-infected aly/aly mice were intraperitoneally injected with anti-CD25 or anti-FR4 mAb at 26 WPI, and parasite burden in the spleen (A) and bone marrow (B) was estimated by qPCR after 10 days of antibody treatment. * p<0.05; ** p<0.01. (PDF) [file pntd.0001798.s003.pdf]

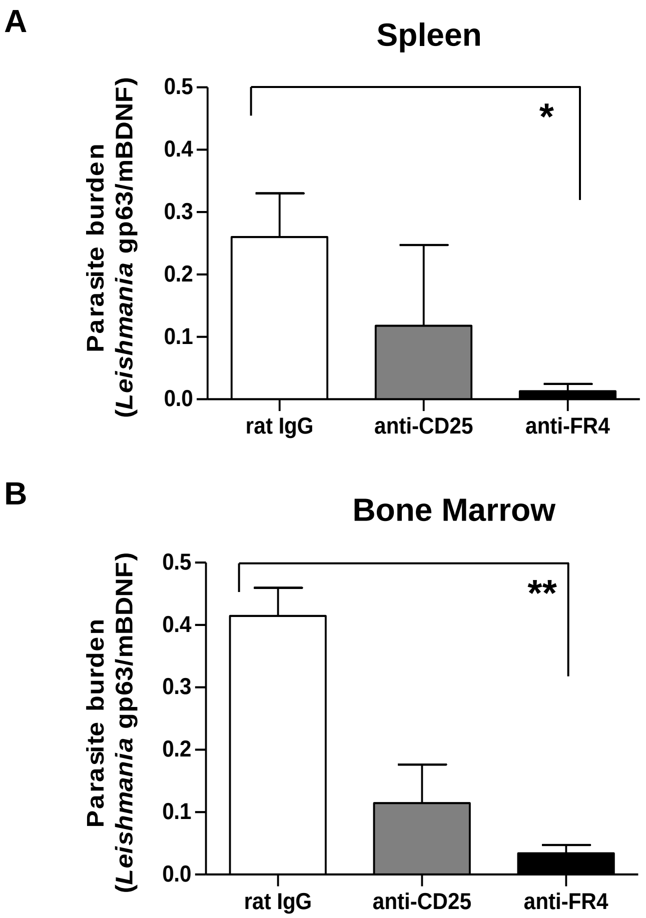

Figure S3. **Effects of anti-Treg antibody treatment on parasite burden in the spleen and bone marrow.**

The *L. donovani*-infected *aly/aly* mice were intraperitoneally injected with anti-CD25 or anti-FR4 mAb at 26 WPI, and parasite burden in the spleen (A) and bone marrow (B) was estimated by qPCR after 10 days of antibody treatment. * $p<0.05$; ** $p<0.01$.
