# Supplementary material for: Involvement of CD4+ Foxp3+ Regulatory T Cells in Persistence of Leishmania donovani in the Liver of Alymphoplastic aly/aly Mice
Source: PLoS Negl Trop Dis. 2012 Aug 21;6(8):e1798. doi: 10.1371/journal.pntd.0001798 (PMC3424244; doi:10.1371/journal.pntd.0001798)
Supplement: Table S1 — Target genes and primers for qPCR and RT-PCR used in this study. (DOCX) [file pntd.0001798.s004.docx]

Table S1. Target genes and primers for qPCR and RT-PCR used in this study

| Gene | Forward primer | Reverse primer | Amplicon size (bp) |
| --- | --- | --- | --- |
| *Leishmania* *gp63* | AGTACGGCTGCGACACCTTGGAG | GTTCCGGCCCCACGGCATCACC | 214 |
| *mBDNF* | CTGGATGCCGCAAACATGTC | CTGCCGCTGTGACCCACTC | 105 |
| *GAPDH* | TCACCACCATGGAGAAGGC | GCTAAGCAGTTGGTGGTGCA | 168 |
| *TGF-β1* | TGACGTCACTGGAGTTGTACGG | GGTTCATGTCATGGATGGTGC | 170 |
| *Foxp3* | CCCAGGAAAGACAGCAACCTT | TTCTCACAACCAGGCCACTTG | 89 |
| *IL-10* | GGTTGCCAAGCCTTATCGGA | ACCTGCTCCACTGCCTTGCT | 191 |
